# PrP^Sc^-Specific Antibodies with the Ability to Immunodetect Prion Oligomers

**DOI:** 10.1371/journal.pone.0019998

**Published:** 2011-05-19

**Authors:** Mourad Tayebi, Daryl Rhys Jones, William Alexander Taylor, Benjamin Frederick Stileman, Charlotte Chapman, Deming Zhao, Monique David

**Affiliations:** 1 Department of Pathology and Infectious Diseases, Royal Veterinary College, Hatfield, Hertfordshire, United Kingdom; 2 National Animal TSE Laboratory, College of Veterinary Medicine, China Agricultural University, Beijing, China; Ohio State University, United States of America

## Abstract

The development of antibodies with binding capacity towards soluble oligomeric forms of PrPSc recognised in the aggregation process in early stage of the disease would be of paramount importance in diagnosing prion diseases before extensive neuropathology has ensued. As blood transfusion appears to be efficient in the transmission of the infectious prion agent, there is an urgent need to develop reagents that would specifically recognize oligomeric forms of the abnormally folded prion protein, PrPSc.

To that end, we show that anti-PrP monoclonal antibodies (called PRIOC mAbs) derived from mice immunised with native PrP-coated microbeads are able to immunodetect oligomers/multimers of PrPSc. Oligomer-specific immunoreactivity displayed by these PRIOC mAbs was demonstrated as large aggregates of immunoreactive deposits in prion-permissive neuroblastoma cell lines but not in equivalent non-infected or *prn-p^0/0^* cell lines. In contrast, an anti-monomer PrP antibody displayed diffuse immunoreactivity restricted to the cell membrane. Furthermore, our PRIOC mAbs did not display any binding with monomeric recombinant and cellular prion proteins but strongly detected PrPSc oligomers as shown by a newly developed sensitive and specific ELISA. Finally, PrioC antibodies were also able to bind soluble oligomers formed of Aβ and α-synuclein. These findings demonstrate the potential use of anti-prion antibodies that bind PrPSc oligomers, recognised in early stage of the disease, for the diagnosis of prion diseases in blood and other body fluids.

## Introduction

Protein aggregates are believed to be the cause of various neurodegenerative disorders, including prion diseases [1]. Soluble oligomeric forms that are recognised in the aggregation process can lead to synaptic dysfunction, whereas large, insoluble deposits are believed to function as reservoirs of the bioactive oligomers [1]. Furthermore, in Alzheimer's disease (AD) and Parkinson's disease (PD), oligomeric forms of amyloid β and -synuclein respectively are believed to form in early phases of diseases and are present in blood and other tissues [2], [3].

The apparent lack of useful specific immune responses is considered a hallmark of prion diseases. Several studies have failed to demonstrate detectable immune responses during the natural course of prion disease reflecting in part the widespread expression of the normal cellular prion protein and the identical primary structure of PrPC and PrPSc leading to B and/or T cell tolerance of disease-associated isoform [4], [5].

Anti-PrP monoclonal antibodies have successfully been raised using various protocols through immunizing *Prn-p^0/0^* mice [6]–[14]. However, only few antibodies have so far displayed the ability to recognize the native non-denatured forms of PrP probably due to the fact that these native proteins lack the capacity to stimulate an immune response in experimental animal models [4], [7], [9], [15]–[18].

In previous work, we showed that immunization of mice with native PrP-coated microbeads led to a mono-specific IgM polyclonal immune response with binding restricted to a motif between PrP amino acids 101–120, [19]. After we demonstrated immunodominance of this specific motif of native PrPSc, Jones and colleagues successfully used PrP peptides derived from this region to produce PrPSc-specific antibodies [11].

In this study, and following immunization of *Prn-p^0/0^* mice with native PrP-coated microbeads, we produced monoclonal antibodies (called PRIOC mAbs) that immunodetect oligomeric forms of native PrPSc as well as other amyloidogenic proteins and peptides. These oligomer-specific mAbs were characterised by ELISA, Western blotting, immunoprecipitation and immunofluorescence imaging and did not display any binding to monomeric recombinant PrP and cellular prion protein in brain tissue of mice as well as monomers and fibrils of other amyloidogenic proteins. All PRIOC mAbs were IgM isotype, consistent with all PrPSc-specific antibodies raised to date by other researchers [8], [11], [20], [21].

PRIOC mAbs could potentially be used for the Immunodetection of soluble oligomeric forms of prions in blood of individuals affected with prion disease and other misfolding diseases.

## Results

### 1. PRIOC mAbs recognise mouse synthetic prion peptides but not monomeric rPrP

**Figure 1:**
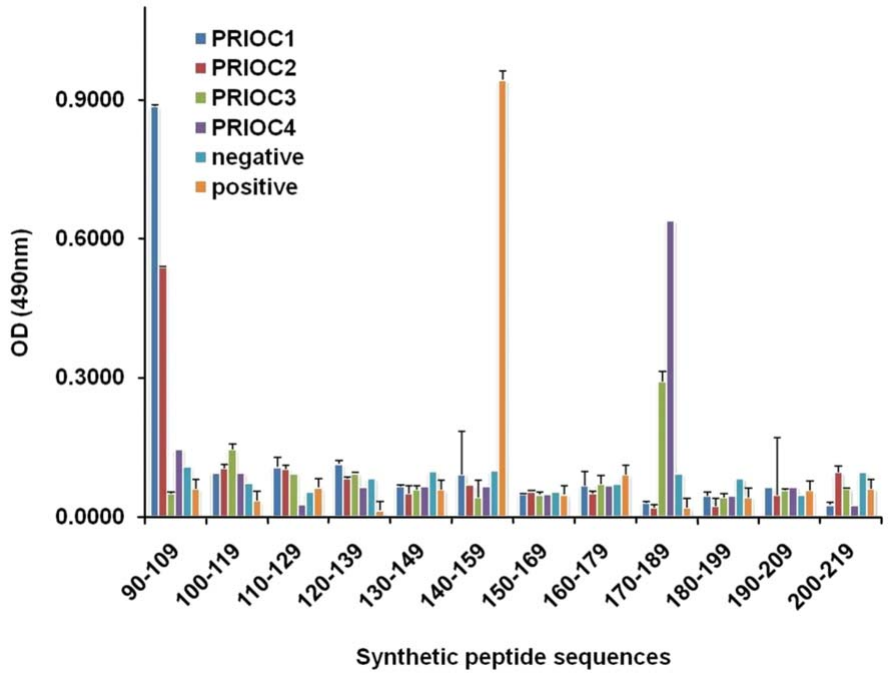
Pep-scan of PRIOC mAbs generated with bead-bound PrPSc. PRIOC1, 2, 3 and 4 mAbs produced in Prn-p0/0 mice immunised with PrP-coated microbeads were investigated using mapping ELISA coated with 20-mer peptides spanning the 90–219 region of the mouse PrP protein. Goat anti-mouse IgM HRP-conjugate was used as secondary detection antibody. An IgG anti-PrP antibody (Sigma) that binds to a region between 143–153 was used as positive control and an antibody isotype-matched control was used as negative control. Anti-PrP responses were measured in peptide ELISA. Values represent the mean ±S.D. of three independent experiments.

Overlapping 20-mer peptides spanning the mouse PrP sequence 90–230 were produced. Depending on the way the immunogen was prepared, the PRIOC mAbs bound different PrP regions. PRIOC2 and PRIOC1 mAbs raised against PrPSc-Dynabeads without prior treatment recognised an amino-terminal epitope between residues 90–109 (Fig. 1). This was identical to the polyclonal anti-sera pep-scan analysis described previously [19].

PRIOC4 mAb appeared to bind to a more C-terminal region of the protein sequence between 170–189. Of note, this region includes the YYR motif used by Paramthiosis and colleagues to raise their PrPSc-specific antibody [21].

Finally, PRIOC3 mAb raised against PrPSc that was first heat-treated before being adsorbed to Dynabeads, weakly bound the 170–189 region of the prion protein similar to that seen with PRIOC4. The results seen with both PRIOC3 and PRIOC4 mAbs are similar to binding seen with the polyclonal antibody response to PrPSc-Dynabeads where the prion-infected brain homogenate was heat treated prior to adsorption to the Dynabeads [19].

PRIOC mAbs were screened for reactivity to full-length and truncated mouse or recombinant prion proteins. None of the mAbs reacted to both and conformations of rPrP with ELISA (data not shown). Furthermore, the isotype of PRIOC mAbs was checked by isotype ELISA and as expected from the polyclonal responses [19], the monoclonal antibody isotype was exclusively IgM (data not shown).

### 2. PRIOC mAbs bind native PrPC/Sc

**Figure 2:**
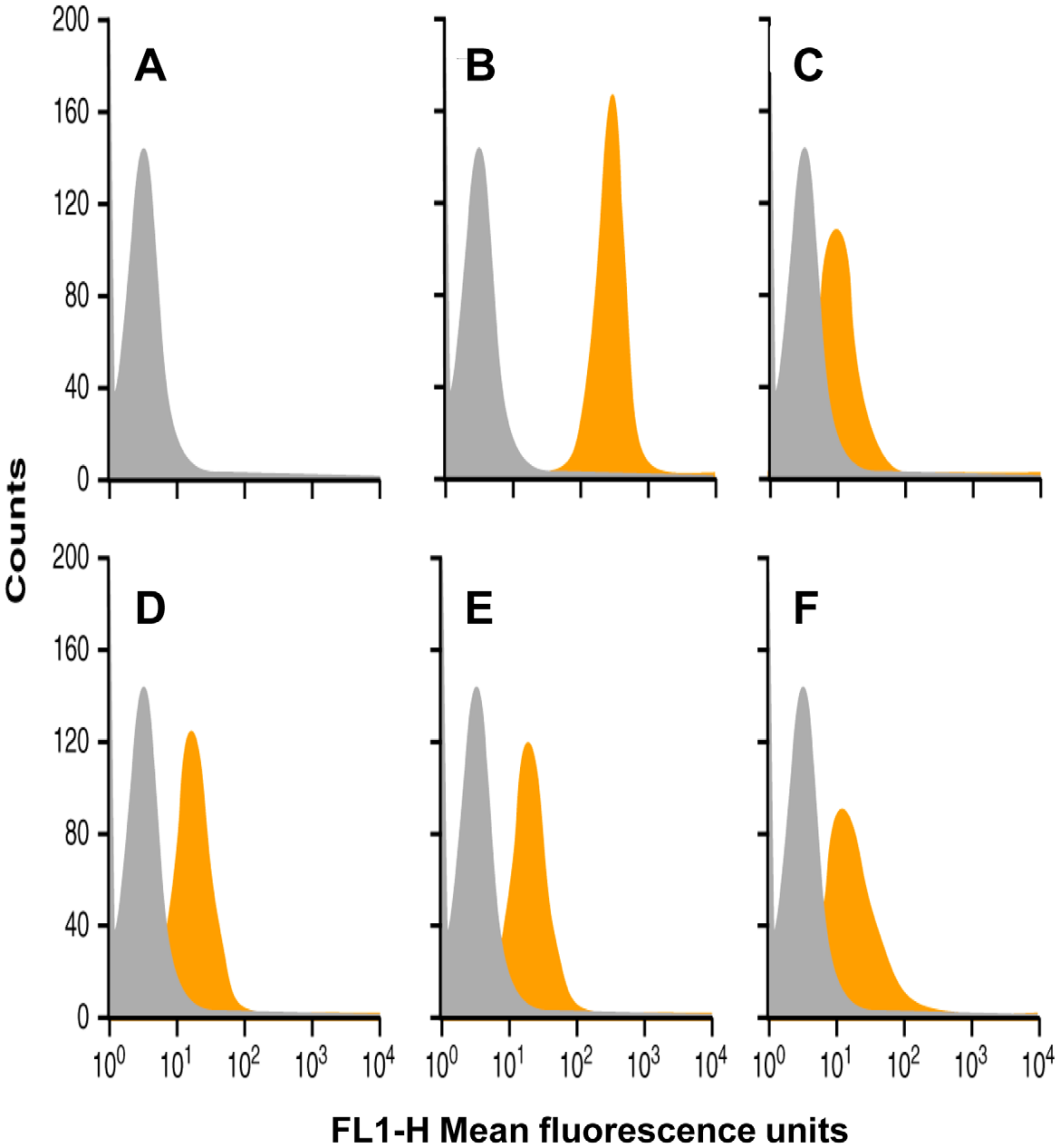
FACS analysis using PRIOC mAbs on ScN2a cells. PRIOC1, 2, 3 and 4 mAbs were used to probe PrP on ScN2a cells. Secondary anti-IgM FITC conjugate was used. Positive and isotype matched FITC conjugate were also used. **A**. cells incubated with anti-IgM FITC conjugate negative control **B**. cells strongly reacted with positive control anti-PrP antibody FITC conjugate **C**. cells reacted poorly with PRIOC1 FITC conjugate **D**. cells reacted mildly with PRIOC2 FITC conjugate. **E**. cells reacted mildly with PRIOC3 FITC conjugate **F**. cells reacted mildly with PRIOC4 FITC conjugate. Representative of three experiments.

Surface PrPC/Sc expression on prion-permissive cell lines, including ScN2a, was shown to be high with anti-PrP antibodies raised against recombinant prion proteins [22]. PRIOC mAbs were also probed in the same way in order to demonstrate whether binding to surface native PrPC/Sc occurred (Fig. 2). In general PRIOC mAbs bound weakly to the surface of ScN2a cells than the anti-PrP positive control (Fig. 2B), possibly reflecting a lower affinity for PrPC/Sc and/or scarcity of oligomeric forms of PrPSc found in these cells.

### 3. PRIOC mAbs bind native murine and human but not native ovine and bovine PrP

**Figure 3:**
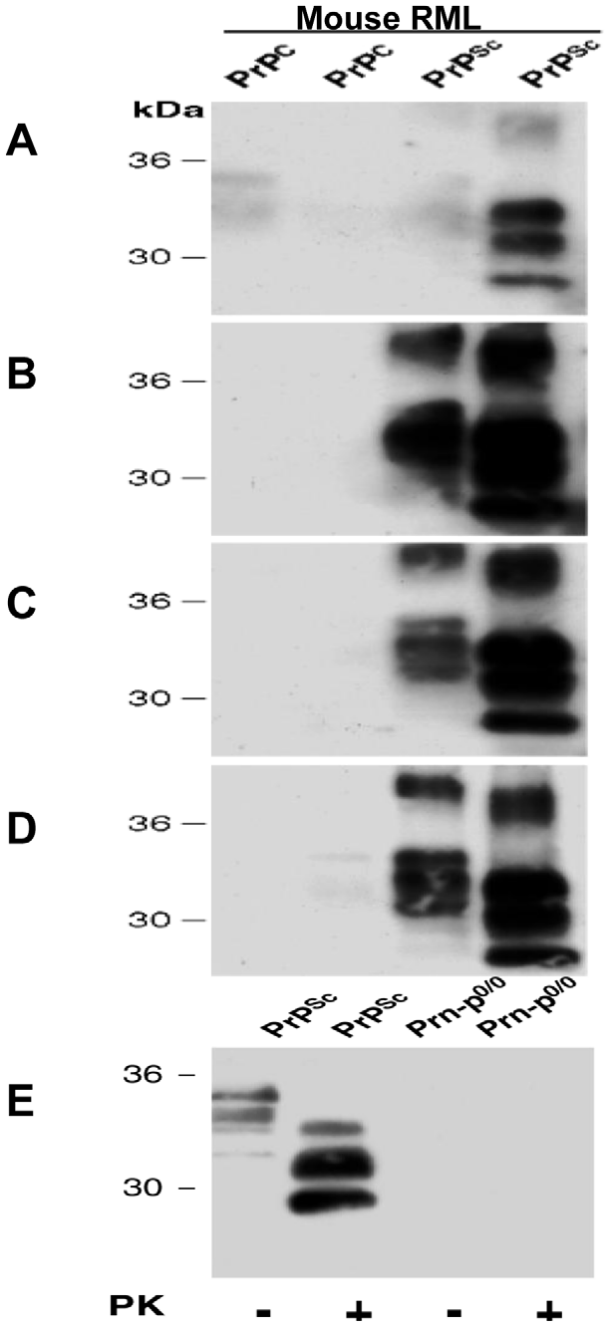
Immunoprecipitation of PRIOC mAbs (5 µg/ml) with murine PrP from brain homogenates (1% w/v). **A**. PRIOC1 immunocapture precipitates from mouse PrPSc but not PrPC with and with no PK treatment **B**. PRIOC2 immunocapture precipitates from mouse PrPSc but not PrPC with and with no PK treatment **C**. PRIOC3 immunocapture precipitates from mouse PrPSc but not PrPC with and with no PK treatment **D**. PRIOC4 immunocapture precipitates from mouse PrPSc but not PrPC with and with no PK treatment **E**. Anti-PrP antibody positive control used to immunocapture precipitates from Prn-p0/0 and PrPSc with and with no PK treatment. Purified PRIOC antibody was used for immunodetection. Representative of three experiments.

**Figure 4:**
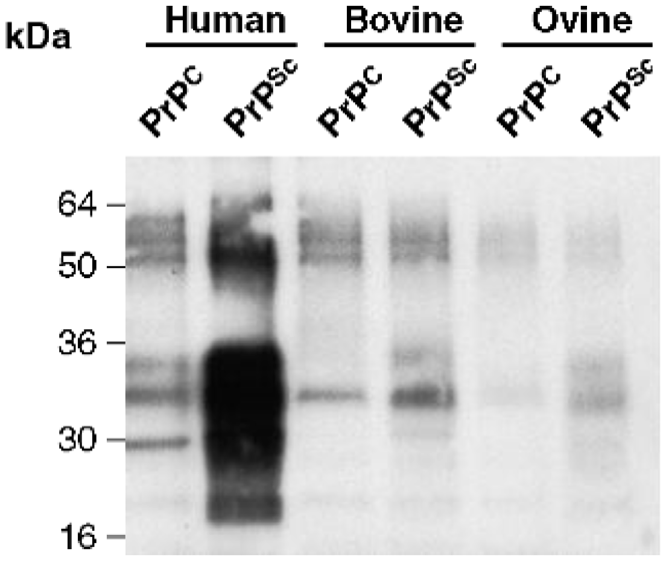
Immunoprecipitation of PRIOC1 mAb (5 µg/ml) with human, bovine and ovine PrP from brain homogenates (1% w/v). PRIOC1 strongly immunocaptures precipitates from human type 4 CJD homogenate but not PrPC with PK treatment. In contrast, PRIOC1poorly immunocaptures precipitates from bovine and ovine PrPC and PrPSc with PK treatment. Representative of three experiments.

Normal and scrapie brain homogenate was proteinase K treated and the PRIOC mAbs used to immunoprecipitate PrPC and PrPSc from murine, human, ovine, and bovine brain homogenates (Table 1). Treatment with proteinase K cleaves approximately 67 amino acids from N-terminus of PrPSc and completely digests PrPC. An anti-PrP antibody (biotin-conjugate), which detects all glycoforms and fragments of PrPC, was used to analyse the immunoprecipitate by western blots after denaturation by boiling in sample buffer for 5 min. After PK treatment, PrPC was completely digested, whereas the 33–35 kDa form of PrPSc was shortened to 27–30 kDa, as a result of degradation of the amino-terminal segment of residues 23–90 analogous to hamster PrPSc [23]. Immunoprecipitation of both control and scrapie-infected brain homogenates with PRIOC mAbs including, PRIOC1, 2, 3 and 4 was performed (Table 1 and Fig. 3 & Fig. 4). The homogenates were proteinase K treated (or not) before incubation with PRIOC mAbs. PRIOC1 mAb bound RML-infected brain homogenates after PK digestion but failed to recognize both mouse and human control brain homogenates (Fig. 3A & Fig. 4). It was also interesting to note that PRIOC1 reacted strongly with type 4 CJD brain homogenates only after PK treatment (data not shown). In contrast, PRIOC2, 3 and 4 bound very strongly to RML-infected brain homogenates and human type 4 CJD whether PK-digested or not (Table 1 and Fig. 3B, C & D) but failed to recognise control homogenate (Fig. 3B, C & D). Of note, all PrioC mAbs failed to display any detectable binding with sporadic CJD (sCJD) with and with no denaturation of the tissue homogenate as tested by Western blotting and immunohistochemistry (data not shown).

**Table 1:** Immunoprecipitation profile of PRIOC mAbs with various brain homogenates.

	*WT*	*WTPK*	*RML*	*RMLPK*	*H*	*HPK*	*T4*	*T4PK*
**PRIOC1**	+	−	+	+++	+	−	+	+++
**PRIOC2**	−	−	+++	+++	−	−	++	++
**PRIOC3**	−	−	+++	+++	−	−	++	++
**PRIOC4**	−	−	+++	+++	−	−	++	++

	*SC*	*SCPK*	*ScC*	*ScCPK*	*BOV*	*BOVPK*	*BSE*	*BSEPK*
**PRIOC1**	−	−	+	+	+	−	+	+
**PRIOC2**	+	−	+	+	−	−	+	+
**PRIOC3**	−	−	−	−	−	−	−	−
**PRIOC4**	+	−	+	+	+	−	+	+

WT (mouse control), RML (scrapie-infected mouse), H (human control), T4 (vCJD), SC (sheep control), ScC (scrapie-infected sheep), BOV (bovine control), and BSE were all immunoprecipitated with PRIOC mAbs with and without proteinase K (PK) prior to immunocapture.

Finally all PRIOC mAbs poorly bound any form of both ovine and bovine brain homogenates (Fig. 4), indicating that the immunogen had a direct influence on species-specificity of these antibodies, but other factors could also be involved.

### 4. PRIOC mAbs immunodetect PrPSc oligomers with immunofluorescence staining

**Figure 5:**
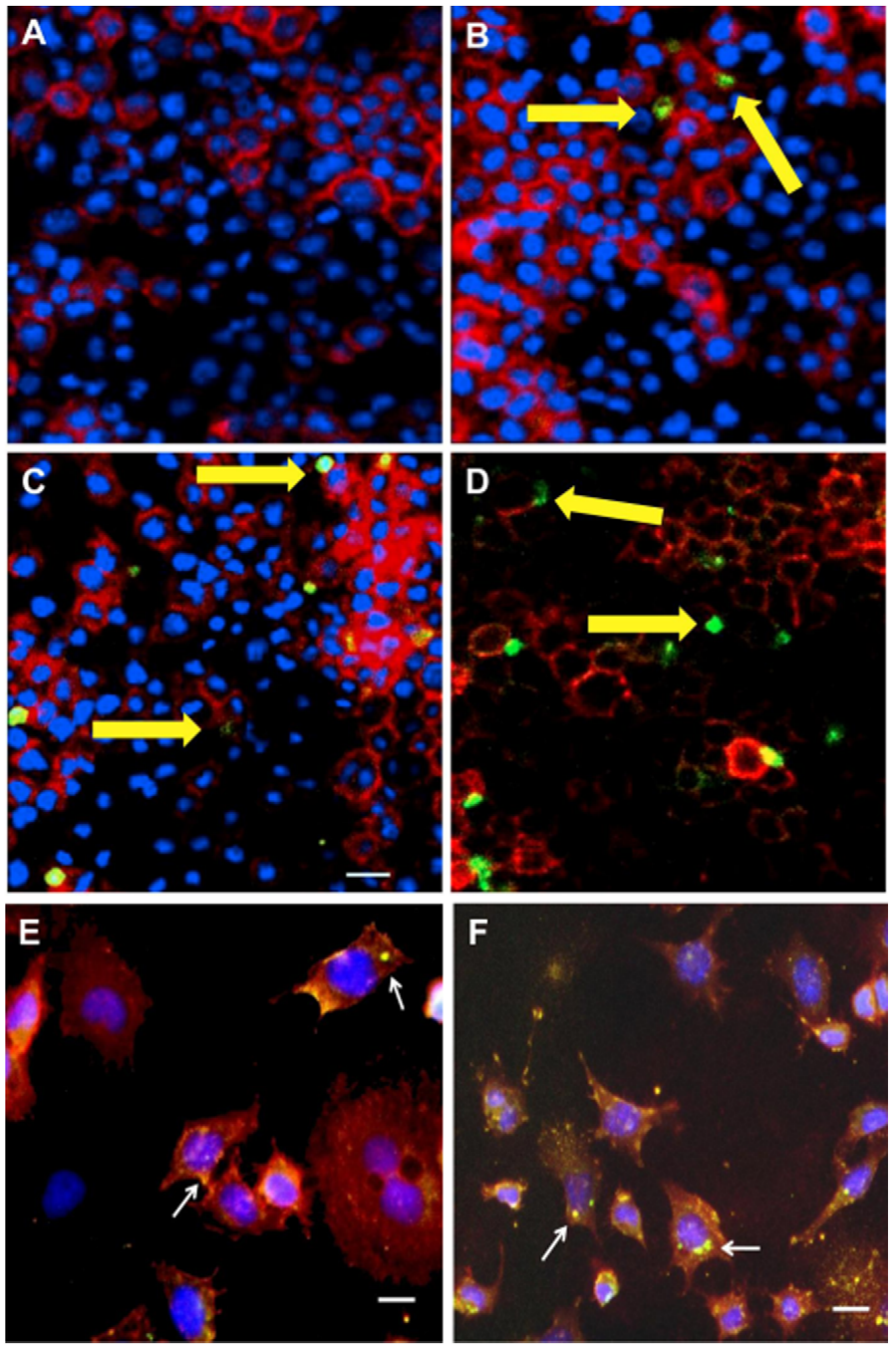
Immunofluorescence staining of PrPSc oligomers/multimers with PRIOC mAbs. PRIOC extensively stained large aggregates in ScN2a cells. (**A**). Staining with PRIOC1 did not display PrPSc oligomer-specific stain. (**B**). PRIOC2, (**C**) PRIOC3 and (**D**) PRIOC4 displayed distinct and unusual staining (yellow arrows) in ScN2a cells (**green**) as opposed to an anti-monomer antibody that displayed the typical diffuse ring-shaped stain of the cell membrane (**red**). Following permeabilization, ScN2a cells displayed similar stain for aggregates using (**E**). PRIOC2 and (**F**). PRIOC2. Florescence microscopy was performed and images from each source [FITC (450–490 nm), Texas red (510–560 nm) and DAPI (330–380 nm)] were collected. Scale bar = 25 µm.

**Figure 6:**
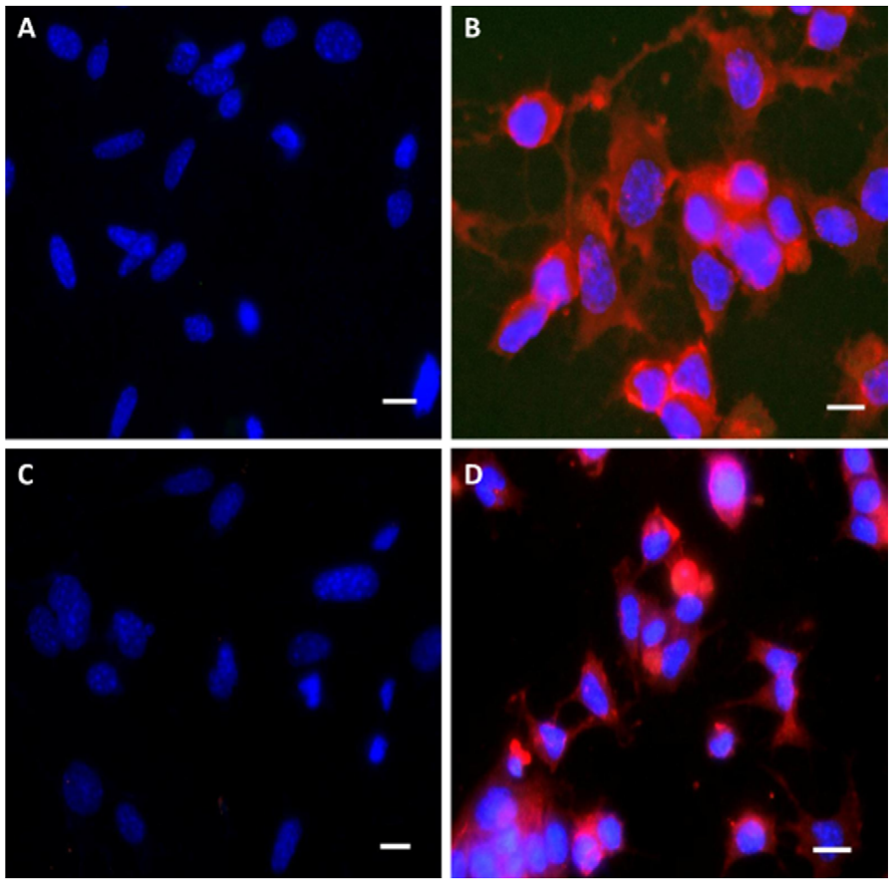
Immunofluorescence staining of N2a and *prn-p^0/0^* cell lines with PRIOC mAbs. PRIOC mAbs failed to stain prion aggregates in N2a and *prn-p^0/0^* glial cells. (**A**). Staining with PRIOC4 did not display PrPSc oligomer-specific stain in *prn-p^0/0^* glial cells with no permeabilization. (**B**). Staining with PRIOC4 did not display PrPSc oligomer-specific stain in N2a cells with no permeabilization. (**C**). Staining with PRIOC4 did not display PrPSc oligomer-specific stain in *prn-p^0/0^* glial cells following permeabilization. (**D**). Staining with PRIOC4 did not display PrPSc oligomer-specific stain in N2a cells following permeabilization. Florescence microscopy was performed and images from each source [FITC (450–490 nm), Texas red (510–560 nm) and DAPI (330–380 nm)] were collected. Scale bar = 25 µm.

The distribution of oligomer-specific immunoreactivity was examined in N2a, ScN2a and a *prn-p^0/0^* glial [24] cell line following binding with PRIOC mAbs (Fig. 5 & Fig. 6). Oligomer-specific immunoreactivity is demonstrated as large aggregates of immunoreactive deposits in ScN2a with (Fig. 5 A, B, C & D) and with no cell permeabilisation (Fig. 5 E & F). In Sharp contrast, anti-PrP antibody positive control displayed the traditional staining pattern focused on the cell membrane decorating it with a ring and appears to be surrounded peripherally by large deposits (Fig. 5). PrioC mAbs failed to bind to both N2a and the *prn-p^0/0^* glial cell, indicating their specific recognition of oligomers associated with prion replication in ScN2a cell lines (Fig. 6). These results suggest that PRIOC mAbs are able to bind a different species of PrPSc; the oligomeric forms, in contrast with the monomers bound by the control antibody.

### 5. PRIOC mAbs immunodetect native PrPSc in ELISA

**Figure 7:**
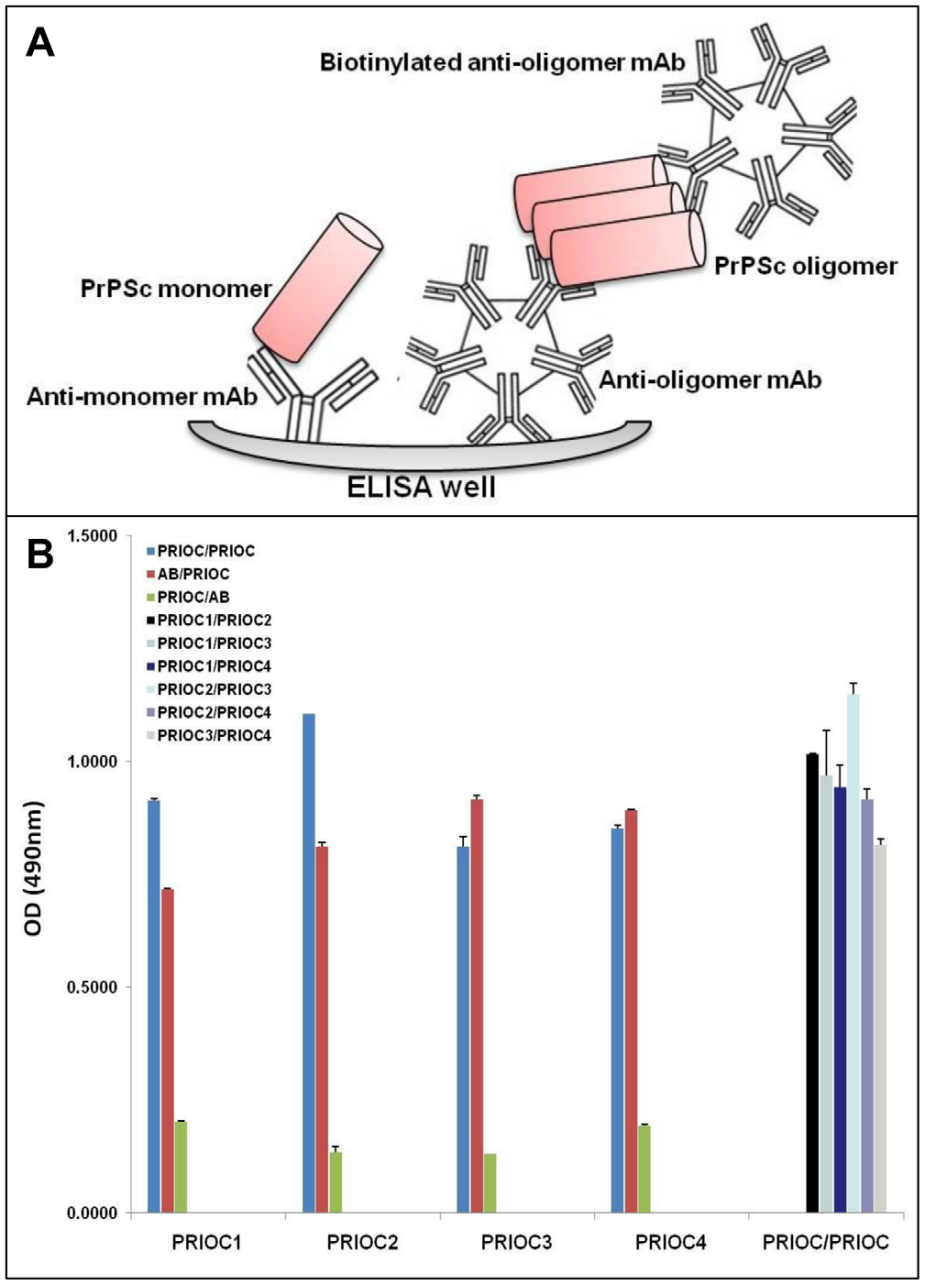
Immunodetection of PrPSc oligomers/multimers with PRIOC mAbs by Sandwich ELISA. (**A**). Principle of a newly developed specific and sensitive ELISA designed for the detection of PrPSc oligomers/multimers. (**B**). RML-infected brain homogenates were used to assess specific binding of PRIOC mAbs to PrPSc oligomers following proteinase K (PK) digestion. 50 µl of 5 µg/ml of PRIOC or anti-monomer antibody (AB) was used to coat the ELISA plate in coating buffer. RML-infected brain homogenate was added to the wells followed by a biotinylated PRIOC mAb or anti-monomer antibody (AB). The sandwich format of the assay has established the specificity of PRIOC antibody for post-PK PrPSc oligomers. Error bars represent the mean antibody level derived from n = 4 wells.

In order to quantify levels of PrPSc oligomers in RML-infected brain homogenates, and to prove the specificity of the PRIOC mAbs in detecting oligomeric forms, we developed a specific and sensitive novel Sandwich ELISA that relies on a non-denaturing protocol designed to immunodetect only the oligomeric species of PrPSc. The assay is based on a Sandwich system that includes an immunocapture PRIOC antibody, able to detect the oligomers, followed by immunodetection with the same PRIOC mAb in a biotinylated form (Fig. 7A). Since PrP monomers are not bound by PRIOC antibody immunocapture, no signal was detected, in contrast, PRIOC antibody binding to oligomers displayed strong signal following immunodetection and substrate stimulation (Fig. 7).

Immunodetection with an anti-PrP antibody able to detect monomeric forms of PrP with subsequent immunodetection with PRIOC antibodies led to a strong positive signal. Of note all PRIOC antibodies detected PrPSc oligomers following capture with anti-monomer antibody (Fig. 7B). In sharp contrast, capture with PRIOC antibodies followed with detection using the anti-monomer antibody failed to give any signal; which strongly indicates that PRIOC mAbs bind to a conformational epitope formed as a consequence of the aggregation process.

### 6. PRIOC mAbs immunodetect rPrP oligomers in ELISA

**Figure 8:**
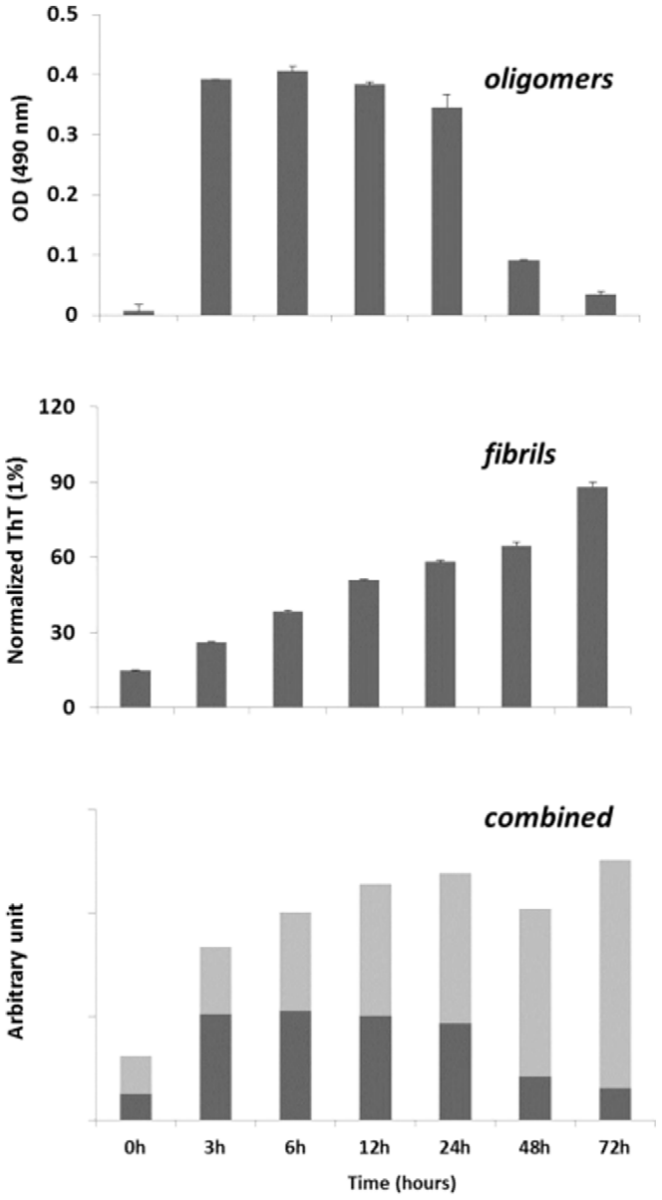
Immunodetection of rPrP oligomers/multimers with PRIOC mAbs by Sandwich ELISA. (**A**). Oligomers derived from rPrP monomers were used to assess specific binding of PRIOC mAbs. 50 µl of 5 µg/ml of PRIOC was used to coat the ELISA plate in coating buffer. rPrP-derived oligomers were added to the wells followed by a biotinylated PRIOC mAb. (B). Fibrils derived from rPrP monomers (used to produce the oligomers) were assayed with thioflavin T fluorescence (Tht). (C). This panel represents a combination of panel (A) and panel (B) and demonstrates the dynamic of oligomer/fibril formation of monomeric rPrP as assessed by the PRIOC mAbs and Tht. Error bars represent the mean level derived from n = 4 wells.

We also tested the ability of PrioC mAbs to detect synthetic oligomers produced from recombinant PrP (rPrP) (Fig. 8). We show that only the oligomeric species derived from monomeric rPrP were immunodetected by PrioC mAbs which failed to bind to both the monomers and the fibrils (Fig. 8A and data not shown). The intensity of Thioflavin T stain of the fibrils derived from monomeric rPrP was inversely proportional to soluble oligomer levels detected by PRIOC mAbs (Fig. 8B), suggesting that the conformational epitope recognized by these mAbs is transitional and its structural availability is phase-specific during the oligomerisation process (8C) [25], [26].

### 7. PRIOC mAbs recognise oligomers derived from monomeric Aβ peptide and α-synuclein

**Figure 9:**
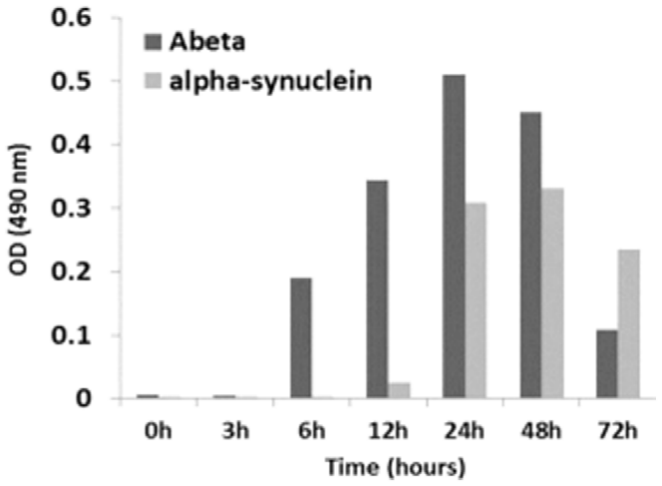
Immunodetection of Aβ peptide and α-synuclein oligomers/multimers with PRIOC mAbs by Sandwich ELISA. Oligomers derived from Aβ peptide and α-synuclein monomers were used to assess specific binding of PRIOC mAbs. 50 µl of 5 µg/ml of PRIOC was used to coat the ELISA plate in coating buffer. Aβ peptide or α-synuclein-derived oligomers were added to the wells followed by a biotinylated PRIOC mAb. Error bars represent the mean antibody level derived from n = 4 wells.

We investigated the ability of PrioC mAbs to bind soluble oligomers derived from monomeric Aβ peptide and α-synuclein by Sandwich ELISA (Fig. 9). All PRIOC mAbs tested were unable to recognize the monomeric isoforms and fibrils but bound strongly to the soluble oligomers derived from Aβ peptide and α-synuclein. Kayed and colleagues have previously shown that oligomer-specific serum raised against Aβ peptide oligomers were able to detect other amyloidogenic proteins and peptides, including α-synuclein, islet amyloid polypeptide (IAPP), polyglutamine, lysozyme, human insulin, and prion peptide 106–126, but failed to recognize either the soluble low-MW species or the fibrils [26]. In agreement, our results indicate that, independently of their primary sequences, a conformational motif common to these amyloidogenic proteins is immunodetected by the PRIOC mAbs.

## Discussion

Previous studies suggest that the toxicity of many amyloidogenic proteins that cause neurodegeneration and neuronal death derive from the soluble oligomeric forms rather than the mature amyloid fibrils believed to act as reservoirs [27]–[30].

The development of reagents that specifically detect these soluble toxic forms in prion disease would be a useful tool to unravel the aggregation process and would help in the diagnosis of the disease by demonstrating the presence of the oligomers in early stages of the disease. To that end, monoclonal antibodies, called PRIOC mAbs were generated in *Prnp^0/0^* mice immunised with RML-infected mouse brain homogenate, corresponding to the protease resistant fragment PrP27–30. These brain homogenates have undergone differential treatment prior to adsorption to the Dynabeads, used as immunogen.

A total of four PRIOC mAbs produced in *PrP^0/0^* mice immunised with PrPSc-Dynabeads, none of which bound to α- or β-rPrP isoforms.

Unexpectedly and except for PRIOC3 that showed weaker binding, All PRIOC mAbs seem to strongly bind linear epitopes as shown by peptide ELISA. Failure of the PrioC mAbs to bind to rPrP while retaining their capability to recognize linear peptides could be explained by the possibility that that PRIOC mAbs are specifically binding to a motif not exposed (or buried) in the recombinant prion protein. Further, binding to a linear peptide does not necessarily mean that the PRIOC mAbs antibodies strictly recognise a linear motif on the prion protein, but more likely bind a conformational epitope as indicated by the competition ELISA with sera from mice immunised with the same immunogen [19]. Furthermore, PRIOC mAbs did not detect native prion proteins by SDS-PAGE electrophoresis with no sample boiling (data not shown), strongly indicating that these antibodies recognise a conformational epitope. This is in agreement with the work of Korth and colleagues [8] where mAb 15B3 recognised three polypetides on the prion protein that were not linear or sequential but were revealed to be contiguous with NMR studies. Williamson and colleagues [31] have also shown that their antibodies mapped “discontinuous epitopes” but bound denatured prion protein.

PRIOC mAbs did not bind native PrPC in mouse control brain homogenates. These results were similar to those shown for binding to control human brain precipitates, where no binding was demonstrated by the PRIOC mAbs. Although all PRIOC mAbs were raised with mouse PrP, cross-reactivity with human PrP, as shown with immunoprecipitation was not surprising as PrP is conserved across species and human and mouse PrP share 98.9% homology [32]. This is also seen with antibodies we raised previously which recognise PrP of all species tested [33]. Similar findings were also observed by other researchers [4], [31], [34].

Heat treatment of RML-infected brain homogenate, prior to adsorption to the Dynabeads, seems to have influenced specific PrP isoform recognition. PRIOC mAbs did not recognise all forms of control mouse and human brain homogeates but also ovine and bovine brain homogenates following immunoprecipitation.

Most of the PRIOC mAbs had similar pattern of binding detecting PrPSc in mouse and human but showed stronger immunodetection of mouse RML as opposed to type 4 CJD.

Molecular classification of human prion diseases has led to the recognition of distinct isolates, or strains [35], [36] with the different fragment sizes seen on Western blots, following proteinase K treatment, suggesting that there are several different human PrPSc conformations. This has also been observed in mink and hamsters [37], [38]. Prion strains can be classified by the ratio of the three PrP bands seen after protease cleavage, corresponding to amino-terminal truncated products generated from di-, mono, or non-glycosylated PrPSc. All PRIOC mAbs have immunodetected RML strain and type 4 CJD with immunoprecipitation and PRIOC2, PRIOC3 and PRIOC4 displayed stronger binding for PrPSc in mouse and human. Treatment of the immunogen prior to immunisation influenced the pattern of binding with various PrP species.

The untreated homogenate that stimulated both PRIOC1 and PRIOC2 demonstrated similar binding pattern as both PRIOC1 and PRIOC2 immunodetected RML-infected brain homogenate and type 4 CJD after PK-digestion. Of note, PRIOC1 did not show binding with either if not subjected to PK digestion. These results demonstrate that differential treatment/binding of the immunogen has not led to dissimilar binding patterns but the observation of different outcomes with some antibodies suggests that the host plays an important role in defining the outcome of the antibody response.

Unsurprisingly, all PRIOC mAbs were of the IgM isotype, similar to the polyclonal responses [19]. All PrPSc-specific antibodies raised to date have been IgM [8], [20], [21], an indication that the immune system uses a pathway that is specific for generating PrPSc-specific antibodies involving perhaps specific B cells that recognise fragments of the prion protein associated with disease only.

PRIOC mAbs were used to assess the distribution of PrPSc immunoreactivity in ScN2a cells by immunofluorescence. Surprisingly, staining with PRIOC mAbs was demonstrated as large aggregates of immunoreactive deposits while staining with an anti-PrP antibody led to a more diffuse pattern forming a ring around the cell membrane. In contrast, these mAbs failed to show any staining with N2a as well *prn-p^0/0^* glial cells. Kayed and colleagues have previously shown that anti-oligomer antibodies revealed that soluble oligomers display a common conformation-dependent structure found only in soluble oligomers independent of their sequences [26]. In agreement with our study, their anti-oligomer antibody displayed a similar binding pattern showing clusters of immunoreactive deposits. Moreover, the seemingly weak binding of PRIOC mAbs with flow cytometry studies can be explained by the scarcity of the aggregates, which indicates that our antibodies recognize PrPSc oligomers. To further prove that PRIOC mAbs immunodetect a conformation-dependent structure formed by the aggregation-oligomerization process, we developed a specific and sensitive Sandwich ELISA that enabled us to detect PrPSc oligomers/multimers, rPrP, Aβ and α-synuclein oligomers but not their monomeric and fibril counterparts.

Immunocapture of PrP oligomers with PRIOC antibodies with subsequent immunodetection with anti-oligomer PRIOC mAbs led to strong signals in contrast with immunodetection with anti-PrP monomer antibody that displayed no signal. The Sandwich assay format used in this study has previously been applied for the detection of α-synuclein oligomers using conformation-dependent anti-α-synuclein oligomers antibody [2], [3]. In agreement, our results displayed similar binding ability of PrPSc oligomers using PRIOC mAbs. Surprisingly, PRIOC mAbs were able to detect soluble oligomers derived from rPrP and other amyloidogenic peptide/protein such Aβ peptide and α-synuclein but failed to display binding to the monomers and fibril isoforms. Further, levels of rPrP oligomers as assessed by PRIOC binding were inversely proportional to fibril formation measured by ThT fluorescence reflecting the dynamic aggregation process of the amyloidogenic proteins.

Taken together our results strongly indicate that these novel PRIOC mAbs raised in *Prn-p^0/0^* mice using native PrP-coated microbeads bind specifically to amyloidogenic oligomers/multimers but not the monomers and the fibrils.

PRIOC mAbs antibodies could potentially be used for the development of a blood-based diagnostic test screen that would detect PrPSc and other oligomers in preclinical prion disease and work is currently underway to achieve this aim.

## Materials and Methods

### 1. Ethics Statement

All procedures involving animals were carried out under a project and personal licence authority issued in accordance with The Animals (Scientific Procedures) Act 1986 and approval by the Royal veterinary College Ethics Committee (approval ID: MT:Towards Immunotherapy for prion diseases, PIL 70/14970). Mice with ablation of both alleles of the single copy PrP gene [39] (Prn-p^0/0^) backcrossed onto an FVB/N strain background (Harlan-Olac UK) were used in the experiments.

### 2. Production of PRIOC monoclonal antibodies

FVB/N *Prn-p^0/0^* mice were immunised subcutaneously with Dynabead-adsorbed PrP in CFA on day 0 then in IFA on days 21, 42, and then finally boosted intraperitoneally on day 50 in PBS instead of adjuvant. PrPSc was first digested with proteinase K (50 µg/ml) for 30 minutes at 37°C to remove PrPC prior to adsorption to the Dynabeads. Hybridomas were screened for reactivity to both native PrPC and PrPSc. Positive hybridomas were repeatedly cloned until stable (Table 2). The isotype of these hybridomas was determined. The IgM-producing hybridomas were unstable when using NS0 cells as fusion partner. Kim and colleagues have shown that SP2/0 myeloma cells express very little PrPC [40]. Using SP2/0 cells as fusion partner had overcome the problem of spontaneous cell death; hence subsequent fusions were performed using this particular myeloma line. Furthermore, the IgM-producing hybridomas were shown to grow better in Dubelco's modified Eagle's medium (DMEM) as compared with RPMI.

**Table 2:** Differential treatment of PrPSc-infected material prior to adsorption to the Dynabeads used to produce PRIOC mAbs.

Fusion partner	Stability	Immunogen	PrP^Sc^ treatment/binding
***PRIOC1***			
NS/0	‘Unstable’	PrP^Sc^-Dynabeads	None
***PRIOC2***			
SP2/0	Stable	PrP^Sc^-Dynabeads	None
***PRIOC3***			
SP2/0	Stable	PrP^Sc^-Dynabeads	Heat-treated
***PRIOC4***			
SP2/0	Stable	PrP^Sc^-Dynabeads	Congo red

All PRIOC mAbs have been generated in *Prn-p0/0* mice immunised with PrP-Dynabeads following treatment with heat, Congo red or no treatment.

### 3. Biotinylation of PRIOC mAbs

The biotinylation procedure used here was a simple method of conjugation using EZ-link Sulpho-NHS-LC-LC biotin (Molecular Weight: 443.43, Pierce). First, PRIOC mAbs were dialysed or buffer exchanged into PBS pH 7.8 (no preservatives). Buffer exchange was performed using Slide-A-Lyzer 10K MWCO Dialysis cassettes (Pierce). In order to achieve efficient labelling, all traces of free amines were removed through at least 5 buffer washes.

Just prior to labelling, 1 mg of biotin was dissolved in 0.5 ml H2O to achieve a 2 mg/ml solution. From the biotin solution, 37 µl volume was added to a 1 mg/ml solution of PRIOC mAb in order to achieve a challenge molar ratio of 20∶1 biotin∶PRIOC. The mixture was then incubated for 1 hour at RT on a rotator. The reaction was stopped by the addition of 40 µl/ml of 2 M glycine. Free unbound biotin was removed through dialysis of the biotinylated antibody against PBS.

Finally, for storage, sodium azide (NaN3) was added to the biotinylated antibody to achieve 0.1% final concentration, as well as trypsin inhibitor (Sigma) to achieve a 2% final concentration. The biotinylated PRIOC mAbs were then aliquoted and stored at −20C.

### 4. Murine synthetic peptides

Peptides were synthesized by automated solid phase step-wise synthesis using the Fmoc N terminal protection chemistry. Peptides were cleaved from the solid phase and fully side-chain deprotected using trifluoroacetic acid with water and tri-isopropylsilane as scavengers. Cleaved peptides were precipitated and washed in ice-cold methyl tertiary butyl ether, dried, dissolved in suitable aqueous solvents and analysed by reverse phase HPLC and MALDI-TOF mass spectrometry. Purified fractions were freeze-dried and then reconstituted in either water or PBS prior to use [19].

### 5. Production of soluble oligomers and fibrils from monomeric rPrP, Aβ peptide and α-synuclein

Soluble oligomers and fibrils derived from their equivalent monomeric isoforms were produced as previously described [26], [41]. Briefly, soluble oligomers were produced by adding 1 mg protein/peptide into 400 µl (hexafluoro-2-propanol) HFIP. The solution (100 µl) was added to 900 µl ddH_2_O and incubated for 10–20 min at RT, which was then centrifuged for 15 min at 14,000× g. The samples were then stirred at 500 rpm for 24 to 48 h at 22°C and 10 µl aliquots were frozen at 6–12 h. The fibrils derived from the monomeric isoforms were prepared as described previously [26]. The Thioflavin T (ThT) fluorescence intensity of the samples was recorded following incubation of 200 µl sample with ThT.

### 6. Enzyme-linked immunosorbent assay (ELISA)

#### 6.1. Epitope mapping with synthetic prion peptides

High binding, 96 well plates (Greiner) were coated with 50 µl/well of a 10 µg/ml peptide solution in coating buffer (35 mM NaHCO3, 15 mM Na2CO3, pH 9.6). The plates were incubated for 1 hour at 37°C then washed 3 times with PBS-0.05% tween 20, and then blocked for 1 h at room temperature. 1 µg/ml of the primary antibody diluted in PBS-0.05% tween 20 was added and incubated for 1 hour at 37°C. The plates were then washed 3 times with PBS-0.05% tween and a 1/1000 dilution of horseradish-peroxidase (HRP) conjugated mouse anti-IgM was added for 25 min at 37°C and the plates were again washed 4 times with PBS-0.05% tween. Finally, the plates were developed with OPD buffer (Sigma) until optimum development occurred, when the reaction was stopped with 3 M sulphuric acid prior to spectrophotometric reading at 490 nm.

#### 6.2. Direct ELISA for detection truncated recombinant PrP

This assay was performed as described above, except that medium binding, 96 well plates (Greiner) were used and coated with 50 µl/well of a 10 µg/ml recombinant protein solution in coating buffer.

#### 6.3. Sandwich ELISA for detection of soluble oligomers

Medium binding, 96 well plates (Greiner) were coated with 50 µl/well of a 5 µg/ml PRIOC antibody solution in coating buffer. The plates were incubated for 1 hour at 37°C then washed 3 times with PBS-0.05% tween, and then blocked for 1 h at room temperature. Brain homogenate diluted to 0.5% in PBS-0.05% tween 20 (w/v) with protease inhibitors (Roche Biochemicals) or 10 µg/ml rPrP, Aβ peptide or α-synuclein was added and incubated for 1 hour at 37°C. The plates were then washed 3 times with PBS-0.05% tween and a 5 µg/ml of biotinylated PRIOC antibody was added for 1 hour at 37°C and the plates were again washed 3 times with PBS-0.05% tween before addition of a 1/1000 dilution of horseradish-peroxidase (HRP) conjugated streptavidin (Sigma) for 25 min at 37°C and the plates were again washed 4 times with PBS-0.05% tween. Finally the plates were developed with OPD buffer until optimum development occurred when the reaction was stopped with 3 M sulphuric acid prior to spectrophotometric reading at 490 nm.

### 7. Immunoprecipitation of PrPSc from various species

Brain tissues from scrapie-infected wild type FVB/N mice were homogenised (10% w/v in PBS) using an Ultra Turrax tissue homogeniser (SIS), and centrifuged for 15 min at 1000× g. In other experiments, brain tissues from human, bovine and ovine were processed in the same way. The supernatants were stored at −80°C until use. Homogenates were diluted to 0.5% in complete Lysis-M buffer with Pefabloc SC plus protease inhibitors (Roche). The mixture was then incubated (1∶1 dilution) with PRIOC antibody for 2 h rotating continuously at 4°C. The immune complexes were then adsorbed overnight to 1×10^6^ anti-IgM-Dynabeads (Invitrogen) rotating continuously at 4°C. The Dynabeads were then washed (four times) using wash buffer. The PrP adsorbed mAb-coated Dynabeads were resuspended in Laemmli buffer [42], and heated to 95°C for 5 min. The beads were finally pelleted and the supernatants used for subsequent Western blotting.

### 8. SDS polyacrylamide gel electrophoresis (SDS-PAGE)

SDS-PAGE pre-cast gels (Invitrogen) were used. Samples to be electrophoresed were diluted 1∶1 in 40 µl sample buffer and boiled for 5 min in screw-cap eppendorf tubes. The samples were spun for 5 seconds at 14.000 rpm in a microfuge before being loaded on the gel. The gels were electrophoresed at a constant voltage of 200V for 1 h. Following electrophoresis, gels were blotted onto Invitrolon PVDF (Invitrogen) in the POWER-PAC (BIO-RAD), at 18V for 45 min. Following blotting, the membranes were rinsed in PBS-tween (0.05%) before being transferred to blocking solution for 60 min at room temperature. The membranes were again rinsed in PBS-tween (0.05%) to remove all traces of blocking solution. An anti-PrP mAb (Sigma) raised against the prion peptide 143–153 in goat was added and incubated for 1 h at room temperature. Following 4 washes of 5 min each, the membranes were then incubated in anti-goat IgG HRP-conjugated antibody diluted at 1 in 10,000. The membranes were washed as above and developed using the Hybond-chemiluminescence (ECL) system (GE Healthcare), according to the manufacturer's instructions. Signal development times ranged from 1 second to 30 min.

### 9. Cell culture

Mouse N2a neuroblastoma cultures (ScN2a) were plated at 2–4×10^6^ in Opti-MEM medium [0.5% (W/V) glucose supplemented with 5% fetal bovine serum (FBS), 50 U/ml penicillin, 50 µg/ml streptomycin and 200 mM L-glutamine]. Cultures were maintained at 37C in 5% CO2 with a change of medium every 48–72 hours.

### 10. Immunofluorescence staining and imaging of PrPSc oligomers

For subsequent staining with PRIOC antibodies, N2a, ScN2a or *prn-p^0/0^* glial cells were first seeded on glass coverslips in 35-mm dishes and grown to 50% confluence at 37°C in a humidified atmosphere of 5% CO2/95% on air. Cells were fixed in 300–500 µl cold (4°C) 3.5–4% (w/v) paraformaldehyde (Fisher Scientific) in TBS for 20 mins at RT prior to staining with antibody for 1 hour. Other cells were subjected to Triton X-100 treatment in order permeabize the cell membrane. After washing the coverslips with TBS, 100 µl of blocking buffer [(1% (v/v) FBS, 1% BSA (w/v) in TBS] was added. The coverslips were incubated with 100 µl of 5 µg PRIOC or 1 µg positive anti-PrP antibody control (Sigma) for 1 hour at RT followed by the secondary antibodies diluted in TBS [(anti-mouse IgM FITC-conjugate, Sigma; anti-goat IgG Texas red-conjugate, Sigma)] for 1 hour at RT. After the final wash in TBS, the coverslips were mounted in fluorescence anti-fade solution (Invitrogen) and sealed with clear nail polish to prevent dehydration.

Florescence microscopy was performed with a *Leica DM4000B* microscope. Images from each source [FITC (450–490 nm), and Texas red (510–560 nm)] were collected by a high resolution DC500 colour camera attached. All images are saved digitally using Leica's IM500 Image Manager Database software from the same field-of-view. Images were merged using Photoshop 6.0 (Adobe). Confocal laser scanning microscopy was performed with a *Zeiss LSM510* confocal system on an inverted Zeiss Axio100M. Z-series and snapshot images were collected. Dual scans were merged using Photoshop 6.0 (Adobe).

### 11. Flow cytometry

ScN2a cells were grown to confluence. Appropriate concentrations of these cells (∼10^7^) were aliquoted into FACS tubes (BD) and washed twice with Hank's medium (Sigma) at 1200 rpm for 5 minutes at 4°C. The tubes were transferred onto ice and PRIOC antibody were added and left to incubate for at least one hour. The cells were washed 3 times in Hank's medium and secondary anti-IgM or negative control anti-IgG FITC-conjugate (Sigma) was added subsequently and left to incubate for another hour. Finally, the cells were washed three times in Hank's medium and scanning was performed.

## References

[1] Haass C, Selkoe DJ (2007). Soluble protein oligomers in neurodegeneration: lessons from the Alzheimer's amyloid beta-peptide.. Nat Rev Mol Cell Biol.

[2] El-Agnaf OM, Salem SA, Paleologou KE, Curran MD, Gibson MJ (2006). Detection of oligomeric forms of alpha-synuclein protein in human plasma as a potential biomarker for Parkinson's disease.. FASEB J.

[3] El-Agnaf OM, Salem SA, Paleologou KE, Cooper LJ, Fullwood NJ (2003). Alpha-synuclein implicated in Parkinson's disease is present in extracellular biological fluids, including human plasma.. FASEB J.

[4] Kascsak RJ, Rubenstein R, Merz PA, Tonna-DeMasi M, Fersko R (1987). Mouse polyclonal and monoclonal antibody to scrapie-associated fibril proteins.. J Virol.

[5] Porter DD, Porter HG, Cox NA (1973). Failure to demonstrate a humoral immune response to scrapie infection in mice.. J Immunol.

[6] Zanusso G, Liu D, Ferrari S, Hegyi I, Yin X (1998). Prion protein expression in different species: analysis with a panel of new mAbs.. Proc Natl Acad Sci U S A.

[7] Williamson RA, Peretz D, Smorodinsky N, Bastidas R, Serban H (1996). Circumventing tolerance to generate autologous monoclonal antibodies to the prion protein.. Proc Natl Acad Sci U S A.

[8] Korth C, Stierli B, Streit P, Moser M, Schaller O (1997). Prion (PrPSc)-specific epitope defined by a monoclonal antibody.. Nature.

[9] Horiuchi M, Karino A, Furuoka H, Ishiguro N, Kimura K (2009). Generation of monoclonal antibody that distinguishes PrPSc from PrPC and neutralizes prion infectivity.. Virology.

[10] Cordes H, Bergstrom AL, Ohm J, Laursen H, Heegaard PM (2008). Characterisation of new monoclonal antibodies reacting with prions from both human and animal brain tissues.. J Immunol Methods.

[11] Jones M, Wight D, McLoughlin V, Norrby K, Ironside JW (2009). An antibody to the aggregated synthetic prion protein peptide (PrP106–126) selectively recognizes disease-associated prion protein (PrP) from human brain specimens.. Brain Pathol.

[12] Polymenidou M, Moos R, Scott M, Sigurdson C, Shi YZ (2008). The POM monoclonals: a comprehensive set of antibodies to non-overlapping prion protein epitopes.. PLoS One.

[13] Demart S, Fournier JG, Creminon C, Frobert Y, Lamoury F (1999). New insight into abnormal prion protein using monoclonal antibodies.. Biochem Biophys Res Commun.

[14] Yokoyama T, Itohara S, Yuasa N (1996). Detection of species specific epitopes of mouse and hamster prion proteins (PrPs) by anti-peptide antibodies.. Arch Virol.

[15] Tayebi M, Collinge J, Hawke S (2009). Unswitched immunoglobulin M response prolongs mouse survival in prion disease.. J Gen Virol.

[16] Nakamura N, Miyamoto K, Shimokawa M, Nishida N, Mohri S (2003). Generation of antibodies against prion protein by scrapie-infected cell immunization of PrP(0/0) mice.. Hybrid Hybridomics.

[17] Spinner DS, Kascsak RB, LaFauci G, Meeker HC, Ye X CpG oligodeoxynucleotide-enhanced humoral immune response and production of antibodies to prion protein PrPSc in mice immunized with 139A scrapie-associated fibrils.. J Leukoc Biol.

[18] Prusiner SB, Groth D, Serban A, Koehler R, Foster D (1993). Ablation of the prion protein (PrP) gene in mice prevents scrapie and facilitates production of anti-PrP antibodies.. Proc Natl Acad Sci U S A.

[19] Tayebi M, Enever P, Sattar Z, Collinge J, Hawke S (2004). Disease-associated prion protein elicits immunoglobulin M responses in vivo.. Mol Med.

[20] Curin SV, Bresjanac M, Popovic M, Pretnar HK, Galvani V (2004). Monoclonal antibody against a peptide of human prion protein discriminates between Creutzfeldt-Jacob's disease-affected and normal brain tissue.. J Biol Chem.

[21] Paramithiotis E, Pinard M, Lawton T, LaBoissiere S, Leathers VL (2003). A prion protein epitope selective for the pathologically misfolded conformation.. Nat Med.

[22] Jones DR, Taylor WA, Bate C, David M, Tayebi M (2010). A camelid anti-PrP antibody abrogates PrP replication in prion-permissive neuroblastoma cell lines.. PLoS One.

[23] Oesch B, Westaway D, Walchli M, McKinley MP, Kent SB (1985). A cellular gene encodes scrapie PrP 27–30 protein.. Cell.

[24] Nishimura T, Sakudo A, Xue G, Ikuta K, Yukawa M (2008). Establishment of a new glial cell line from hippocampus of prion protein gene-deficient mice.. Biochem Biophys Res Commun.

[25] Kayed R, Canto I, Breydo L, Rasool S, Lukacsovich T (2010). Conformation dependent monoclonal antibodies distinguish different replicating strains or conformers of prefibrillar Abeta oligomers.. Mol Neurodegener.

[26] Kayed R, Head E, Thompson JL, McIntire TM, Milton SC (2003). Common structure of soluble amyloid oligomers implies common mechanism of pathogenesis.. Science.

[27] Soto C (1999). Alzheimer's and prion disease as disorders of protein conformation: implications for the design of novel therapeutic approaches.. J Mol Med.

[28] Conway KA, Harper JD, Lansbury PT (2000). Fibrils formed in vitro from alpha-synuclein and two mutant forms linked to Parkinson's disease are typical amyloid.. Biochemistry.

[29] Walsh DM, Klyubin I, Fadeeva JV, Cullen WK, Anwyl R (2002). Naturally secreted oligomers of amyloid beta protein potently inhibit hippocampal long-term potentiation in vivo.. Nature.

[30] Bucciantini M, Giannoni E, Chiti F, Baroni F, Formigli L (2002). Inherent toxicity of aggregates implies a common mechanism for protein misfolding diseases.. Nature.

[31] Williamson RA, Peretz D, Pinilla C, Ball H, Bastidas RB (1998). Mapping the prion protein using recombinant antibodies.. J Virol.

[32] Wopfner F, Weidenhofer G, Schneider R, von BA, Gilch S (1999). Analysis of 27 mammalian and 9 avian PrPs reveals high conservation of flexible regions of the prion protein.. J Mol Biol.

[33] Beringue V, Mallinson G, Kaisar M, Tayebi M, Sattar Z (2003). Regional heterogeneity of cellular prion protein isoforms in the mouse brain.. Brain.

[34] Rubenstein R, Kascsak RJ, Papini M, Kascsak R, Carp RI (1999). Immune surveillance and antigen conformation determines humoral immune response to the prion protein immunogen.. J Neurovirol.

[35] Wadsworth JD, Hill AF, Beck JA, Collinge J (2003). Molecular and clinical classification of human prion disease.. Br Med Bull.

[36] Hill AF, Joiner S, Beck JA, Campbell TA, Dickinson A (2006). Distinct glycoform ratios of protease resistant prion protein associated with PRNP point mutations.. Brain.

[37] Bessen RA, Marsh RF (1992). Biochemical and physical properties of the prion protein from two strains of the transmissible mink encephalopathy agent.. J Virol.

[38] Bessen RA, Marsh RF (1994). Distinct PrP properties suggest the molecular basis of strain variation in transmissible mink encephalopathy.. J Virol.

[39] Bueler H, Fischer M, Lang Y, Bluethmann H, Lipp HP (1992). Normal development and behaviour of mice lacking the neuronal cell-surface PrP protein.. Nature.

[40] Kim JI, Kuizon S, Rubenstein R (2003). Comparison of PrP transcription and translation in two murine myeloma cell lines.. J Neuroimmunol.

[41] Kayed R, Head E, Sarsoza F, Saing T, Cotman CW (2007). Fibril specific, conformation dependent antibodies recognize a generic epitope common to amyloid fibrils and fibrillar oligomers that is absent in prefibrillar oligomers.. Mol Neurodegener.

[42] Laemmli UK (1970). Cleavage of structural proteins during the assembly of the head of bacteriophage T4.. Nature.

